# Aberrant super-enhancer landscape reveals core transcriptional regulatory circuitry in lung adenocarcinoma

**DOI:** 10.1038/s41389-020-00277-9

**Published:** 2020-10-17

**Authors:** Te Zhang, Xuming Song, Zeyu Zhang, Qixing Mao, Wenjie Xia, Lin Xu, Feng Jiang, Gaochao Dong

**Affiliations:** 1 Department of Thoracic Surgery, Affiliated Cancer Hospital of Nanjing Medical University, Jiangsu Cancer Hospital, Jiangsu Institute of Cancer Research, Jiangsu Key Laboratory of Molecular and Translational Cancer Research, Nanjing, 210009 China; 2 The Fourth Clinical College of Nanjing Medical University, Nanjing, China

**Keywords:** Non-small-cell lung cancer, Oncogenes

## Abstract

Lung adenocarcinoma (LUAD) relies on dysregulated gene expression to sustain its infinite growth and progression. Emerging evidence indicates that aberrant transcriptional program results from core transcriptional regulatory circuitry (CRC) which is driven by super-enhancers (SEs). In this study, by integrating profiles of H3K27Ac chromatin immunoprecipitation sequencing (ChIP-seq) from normal adult lung and LUAD cell lines, we revealed that widespread alterations of the super-enhancer were presence during lung carcinogenesis. With SE-based modeling of regulatory circuits and assessments of transcription factor (TF) dependencies, we reconstructed an interconnected transcriptional regulation network formed by three master TFs, including ELF3, EHF, and TGIF1, all of which promoted each other’s expression that confirmed by ChIP-qPCR and western blot. Loss-of function assay revealed that each of them is essential for LUAD cells survival, invasion and metastasis. Meanwhile, the rescue assay also illustrated the transacting transcriptional regulatory circuitry. In addition, the mRNA levels of ELF3, EHF, and TGIF1 were differentially expressed in LUAD tumors and peritumoral tissue. IHC of serial sections revealed that high expressions of CRC (ELF3/EHF/TGIF1-High) were closely associated with high proliferative activity in tumor tissue and poor prognosis on patients with LUAD. Finally, we used small molecular inhibitors to perturb the transcriptional circuitry, also exhibited a prominent anti-cancer effect in vitro. Our findings reveal the mechanism of the transcriptional dysregulation and addiction of LUAD.

## Introduction

Lung cancer is the leading cause of cancer-related death worldwide because of its high incidence and associated mortality^1^. In recent years, lung cancer has a substantial mortality rate and the incidence of lung cancer has been increasing gradually^2^. According to histological features, lung cancer is divided into small cell lung cancer (SCLC) and non-SCLC (NSCLC). NSCLC accounts for over 80% of all lung carcinomas and continues to increase in incidence^3^. There are two main subtypes of NSCLC: lung adenocarcinoma (LUAD) and squamous cell carcinoma (LUSC), of which LUAD is the most common. With a 5-year survival rate of only 10%, it remains of great importance to explore the underlying mechanisms of LUAD to develop more effective therapeutic interventions.

Transcriptional dysregulation is a prominent hall-mark of cancer. The *cis*-regulatory elements known as enhancers are key modulators of cell type-specific expression programs. Recently, several independent groups reported that large regions with clustered enhancer units, termed super-enhancers (SEs), have been identified^4^. SE was used to characterize large genomic domains, playing a key role in regulating the expression of key cell identity^5^, and promoting oncogenic transcription to which cancer cells become highly addicted^6,7^. Breast cancer could evolve SE to drive CD47 overexpression to escape immune surveillance^8^. The overexpression of SE-driven EVT6 was clinically associated with poor prognosis in patients with nasopharyngeal carcinoma^9^. In adult T-cell leukemia, SE is of great help for the identification of critical cancer genes^10^. Targeting SE-associated oncogenes could suppress tumor progression in esophageal squamous cell carcinoma^11^, colon cancer^12,13^, MDS-related leukemia^14,15^, and rhabdomyosarcoma^16^. Currently, there is no report regarding the association of SE with LUAD thus far.

SE recruits an exceptionally large number of transcription factors (TFs) and cofactors. The interconnected auto-regulatory loop, like core regulatory circuitry (CRC)^17^, was established among SE-associated master TFs^18^. CRC was identified in a variety of tumors, such as neuroblastoma^16,19^, chronic lymphocytic leukemia^20^, T cell acute lymphoblastic leukemia^21^, gastrointestinal stromal tumor^22^, urinary bladder carcinoma^23^, and liposarcoma^24^. However, no prior research has investigated the landscape of CRC and associated targeted therapeutics in LUAD.

Here, we compared the SE-landscapes between normal lung tissues and LUAD cells and identified the core transcriptional regulation network formed by three master TFs, including ELF3, EHF, and TGIF1. Concomitantly, the master TFs network was confirmed by chromatin Immunoprecipitation polymerase chain reaction (ChIP-PCR) and rescue experiments. Finally, inhibition of transcriptional regulation network by small molecular inhibitors suppressed tumor survival which provided a therapeutic strategy for LUAD treatment.

## Materials and methods

### Super enhancer analysis

H3K27ac ChIP-seq data and control of normal adult lung and LUAD cell line (A549 and PC-9) were obtained from the ENCODE project (http://www.encodeproject.org). The control libraries of normal adult lung are ENCLB721DHS and ENCLB763VML, while the H3K27ac ChIP-seq libraries are ENCLB163YYP and ENCLB779TPX. The control library of A549 cell line is ENCLB695ADF and the H3K27ac library is ENCLB695AFP. The control library of PC-9 cell line is ENCLB539LMN and the H3K27ac library is ENCLB187KMZ. Model-based analysis of ChIP-Seq data were utilized to identify the regions of H3K27ac ChIP-seq enrichment and ROSE2 algorithm was used to define SEs. The distance of constituent enhancers was fixed at 12.5 kb. Target genes of super enhancers were obtained from the ROSE2 ENHANCER TO TOP GENE.txt file.

### Core transcriptional regulatory circuitry

CRCmapper_2017 was used to construct the core transcriptional regulatory circuitry. FIMO was installed through the conda from the MEME, also SAMtools was installed. TFlist_NMid_hg.txt was download as well as the CRCmapper_2017.Hg19_ref_seq.ucsc was used to annotate the genome files.

### TCGA and Pan-cancer analysis

LUAD of TCGA was download from UCSC database (http://xenabrowser.net). DESeq2 R package was used to analyze the different gene expression. The Kaplan–Meier analysis was performed via Kaplan–Meier Plotter (http://www.kmplot.com/lung/). Furthermore, we obtained the gene expression profiles of ELF3, EHF, and TGIF1 in normal and tumor tissues from gene expression profiling interactive analysis (GEPIA; http://gepia.cancer-pku.cn/index.html), using a standard system to perform pan-cancer analysis.

### Cell cultures

Human LUAD cell lines PC-9 and A549 were purchased from KeyGen Biotech (KeyGen Biotech, Nanjing, China), and detected for mycoplasma before use. Cells were maintained in Dulbecco’s modified eagle medium or RPMI 1640 medium (HyClone, Utah, USA) supplemented with 10% heat-inactivated fetal bovine serum (FBS; Gibco, Newcastle, Australia), at 37 °C in an incubator containing 5% CO_2_. The identity of all cell lines was recently verified by short tandem repeat analysis in 2019.

### Transfections

Lipofectamine RNAiMax reagent (Thermo Fisher) was used for siRNAs transfections. Briefly, 2 × 10^4^ PC-9 cells were subjected to double pulse of reverse-transfection by using 4 μl of Lipofectamine RNAiMax and cells were collected or re-plated for further experiments 24 h after the last pulse of transfection. Plasmid transfections were performed on 80% confluent cells by using Lipofectamine 3000, following manufacturer’s instructions (Thermo Fisher). ELF3, EHF, and TGIF1 plasmids were bought from PPL (Public Protein/Plasmid Library, China). siRNAs are listed in Table S1. Plasmids are listed in Table S2.

### Small molecule Inhibitors

JQ1, OTX015, SGC-CBP30, and THZ1 were purchased from APExBIO (APExBIO Technology LLC, Houston, USA).

### Transwell migration and invasion assays

For migration assay, cells were plated into the upper chamber (8 μm; Corning, Tewksbury, USA). For invasion assay, cells were added into the upper chamber precoated with Matrigel (8 μm; Corning, Tewksbury, USA). The lower chamber was filled with RPMI-1640 supplemented with 20% FBS. The membranes were incubated for 24–48 h and then were stained with 0.1% crystal violet for 10 min. The numbers of migrated and invaded cells on lower surface of the membrane were calculated using a microscope (Olympus, Tokyo, Japan).

### Wounding assay

PC-9 cells (4 × 10^5^ cells per well in 12-well plates) were transfected with siRNAs (Scramble, siELF3-pool, siEHF-pool or siTGIF1-pool). At 24 h after transfection, cell monolayers were wounded by scratching with sterile plastic 200 μl micropipette tips. Cells were photographed using phase-contrast microscopy: immediately and 24 h after wounding. The assay was independently performed in triplicate. The migration area was measured by graphic software Adobe Photoshop (Adobe, San Jose, CA, USA).

### 3D tumor spheroids culture

PC-9 cells were cultured as hanging drops for 24 h to form spheroids. Each spheroid contained approximately 5000 cells. Cells were seeded into gelatin-functionalized non-adherent U bottom 96-well plates (4515, Corning, New York, USA). Cells grew into spheroids for 5 days at 37 °C, 5% CO_2_. Images were collected using an Leica (DM3000B) microscope.

### Western blotting

For protein extract preparation, cells were lysed on ice with RIPA Lysis Buffer (ThermoFisher) containing complete protease and phosphatase inhibitor cocktail (Roche). Soluble protein extracts were separated by centrifugation at 13000 rpm for 15 min and diluted in Laemlli sample buffer. The obtained cell lysates were resolved on sodium dodecyl sulfate-polyacrylamide gels electrophoresis (SDS-PAGE) and transferred on polyvinylidene difluoride membrane Hybond TM-P (Amersham Bioscience). Membranes were saturated with 5% bovine serum albumin at room temperature for 2 h and incubated with the following primary antibodies at 4 °C overnight. Primary antibodies were used as follows: ELF3 (1:1000, AF5787; R&D Systems, Minneapolis, USA), EHF (1:1000, 27195-1-AP; Proteintech, Illinois, USA), TGIF1 (1:1000, ab52955; Abcam, Masseachusettes, USA), β-actin (1:1000, 3700S; CST, Boston, USA) and GAPDH (1:1000, 2118S; CST, Boston, USA). Secondary anti-mouse IgG (ab175775), anti-rabbit IgG (ab175773), and anti-goat IgG (ab175776) all conjugated to Alexa Fluor 680 (Abcam, Masseachusettes, USA) were incubated with the membranes for 2 h at room temperature at 1:10,000 dilution. All bands of western blot were detected and qualified with gray scale ratio by Odyssey CLx imaging systems (LI-COR, Nebraska, USA).

### Immunohistochemistry

Immunohistochemistry (IHC) was performed on paraffin-embedded tumor and peritumoral tissues. After fixation in 4% paraformaldehyde (Invitrogen), 7 μm paraffin slides were rehydrated and treated with hydrogen peroxide. Antigen retrieval was induced by heat in Tris-EDTA (pH9). The sections were incubated with a specific antibody and diaminobenzidine (Thermo Fisher) was used as a detection method followed by hematoxylin counterstaining. Antibody as follows: anti-Ki67 (1:200, ab15580; Abcam), anti-TGIF1 (1:200, sc-17800; Santa Cruz), anti-ELF3 (1:200, MAB57871; R&D Systems), and anti-EHF (1:200, PA5-63890; Invitrogen).

### Chromatin immunoprecipitation quantitative polymerase chain reaction (ChIP-qPCR)

ChIP for ELF3, EHF, and TGIF1 were performed using the protocol described previously. Briefly, LUAD cells were cross-linked with a final concentration of 1% formaldehyde (Sigma-Aldrich, St. Louis, USA), and were quenched by a final concentration of 125 mM glycine. After cells were lysed, cold shearing buffer containing protease inhibitors (Roche Applied Science, Mannheim, Germany) was added, and then the chromatin was sheared through sonication to obtain fragment sizes 150–1000 bp. The antibody against ELF3 (R&D Systems, Minneapolis, USA), EHF (Proteintech, Illinois, USA), TGIF1 (Abcam, Masseachusettes, USA), normal mouse IgG (CST, Boston, USA), normal rabbit IgG (CST, Boston, USA), and normal goat IgG (CST, Boston, USA) were used for immunoprecipitation. qPCR (primers were shown in Table S3) was conducted to measure the enrichment of ELF3, EHF, and TGIF1 on DNA molecules of interest.

### Clonogenic assay

For clonogenic assay, single-cell suspensions were planted in 35 mm plates at low density (1200 cells/plate). The medium was changed every 48–72 h. After 9 days, cells were fixed in methanol for 10 min and stained at 4 °C overnight with 0.1% crystal violet solution. Plates were then washed twice with phosphate-buffered saline and dried. Pictures were acquired using digital camera to count the colonies. Results represent the mean ± standard deviation (SD) of three experiments.

### Statistical analysis

All statistical data were displayed as means ± SD and were analyzed using SPSS 20.0 (IBM, Chicago, USA). The significance of the differences between two groups analyzed with Student’s *t* test, while the differences among three or more groups was conducted by using one-way analysis of variance. Differences between the clinicopathological data of master-TFs-high and master-TFs-low patients were analyzed using the chi-square test. All plots were drawn by using GraphPad Prism 6.0 Software (La Jolla, CA, USA). *p < 0.05; **p < 0.01; ***p < 0.001.

### Clinical specimens and measurement

Human tissue study was approved by the Institutional Review Board of Affiliated Cancer Hospital of Nanjing Medical University. All procedures were undertaken in accordance with guidelines set forth by Declaration of Helsinki. Written informed consent was obtained from all of the patients. Fresh cancer and adjacent normal tissue were snap-frozen in liquid nitrogen immediately after surgery and stored in liquid nitrogen. The demographic and clinicopathological details of subtotal 20 LUAD patients were indicated in Table S4.

## Results

### The SE landscape in LUAD is significantly different from that of normal adult lung tissue

**Fig. 1:**
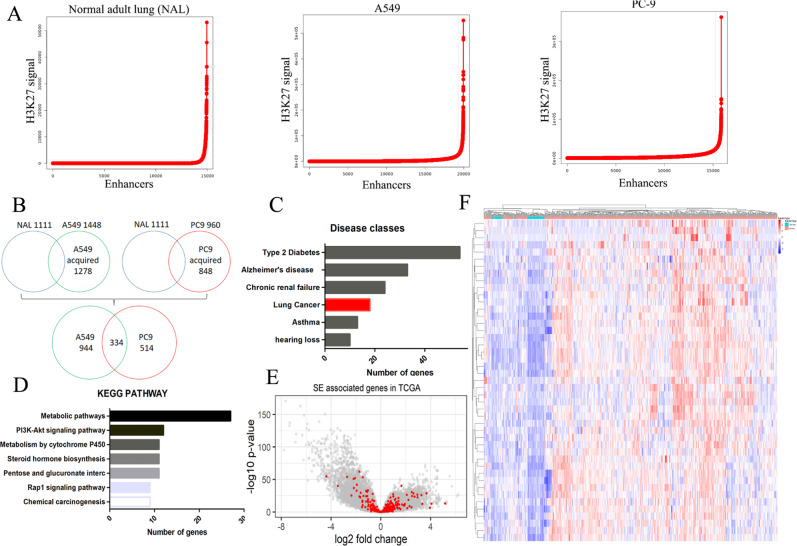
The super-enhancer landscape of LUAD cells differed significantly from that of the normal adult lung. **A** Enhancer regions of the normal adult lung, A549, PC-9 cells were plotted in increasing order based on their H3K27Ac ChIP-Seq signal. Enhancers above the inflection point of the curve were defined as SEs. Examples of SE-genes are listed along with their respective rank. **B** SE-associated gene in A549 and PC-9 cells were systematically compared with SE-associated genes in normal lung tissue. Totally, 1278 and 848 SE-associated genes were acquired in A549 and PC-9 cells, respectively. A total of 1792 SE-associated genes were acquired in A549 and PC-9 cells, in which 375 SE-associated genes were commonly acquired in both cell lines. **C** The gene ontology enrichment analysis suggested that LUAD-SE genes were significantly associated with biological processes essential to cancer sustainability. **D** The KEGG pathway enrichment analysis revealed that LUAD-SE genes were significantly enriched in multiple cancer-relating signaling pathways. **E** Volcano plot illustrating the biological and statistical significance of LUAD-SE genes are highlighted in red, and non-SE-genes are highlighted in gray. **F** The expression profile of the common LUAD-SE gene was clearly segregated between LUAD samples and NT samples in the unsupervised clustering analysis.

To investigate the implications of SE alteration in LUAD, we compared the SE-landscapes between normal adult lung tissue and two LUAD cell lines, A549 and PC-9. We generated a catalog of SEs from the normal adult lung, and the A549, and the PC-9 cells based on the publicly available ChIP-seq data from ENCODE^25,26^. Stitched enhancers which occurred the inflection point of the H3K27ac signal were regarded as SEs (Fig. 1A). To further investigated the alterations of the SE-landscape in LUAD, we compared the SE-associated gene set between normal adult lung tissue and individual canonical LUAD cell lines. We used ChIP-seq data for screening by comparing normal adult lung and LUAD cell lines datasets. A total of 1792 LUAD-acquired SE-associated genes were identified in the LUAD cells, of which 334 genes were commonly acquired in both cell lines (Fig. 1B).

Next, the DAVID pathway enrichment analysis was conducted in 1792 LUAD-acquired SE-associated genes to clarify the biological implications, which showed that these genes were associated with “Lung Cancer” (Fig. 1C). In addition, the result of KEGG pathway enrichment analysis of these genes showed highly enriched in key cancer-associated signaling pathways (Fig. 1D). These results indicated that 1792 genes selected from ChIP-seq dataset by SE identification were exactly involved in LUAD carcinogenesis. Furthermore, we evaluate fold difference in LUAD tissue gene expression relative to adjacent normal lung tissue in TCGA database, which showed that 1792 LUAD-acquired SE-associated genes were higher in LUAD tissue than in normal lung tissue (Fig. 1E). And we performed an unsupervised clustering analysis, suggested that the expression profiles of these SE genes clearly segregated LUAD from non-tumorous lung tissue samples (Fig. 1F). Collectively, these results support a key role for *cis*-regulatory SE at oncogenes, which are acquired in both LUAD cell lines.

### Master TFs in CRC are overexpressed in LUAD and are correlated with poor prognosis in LUAD patients

**Fig. 2:**
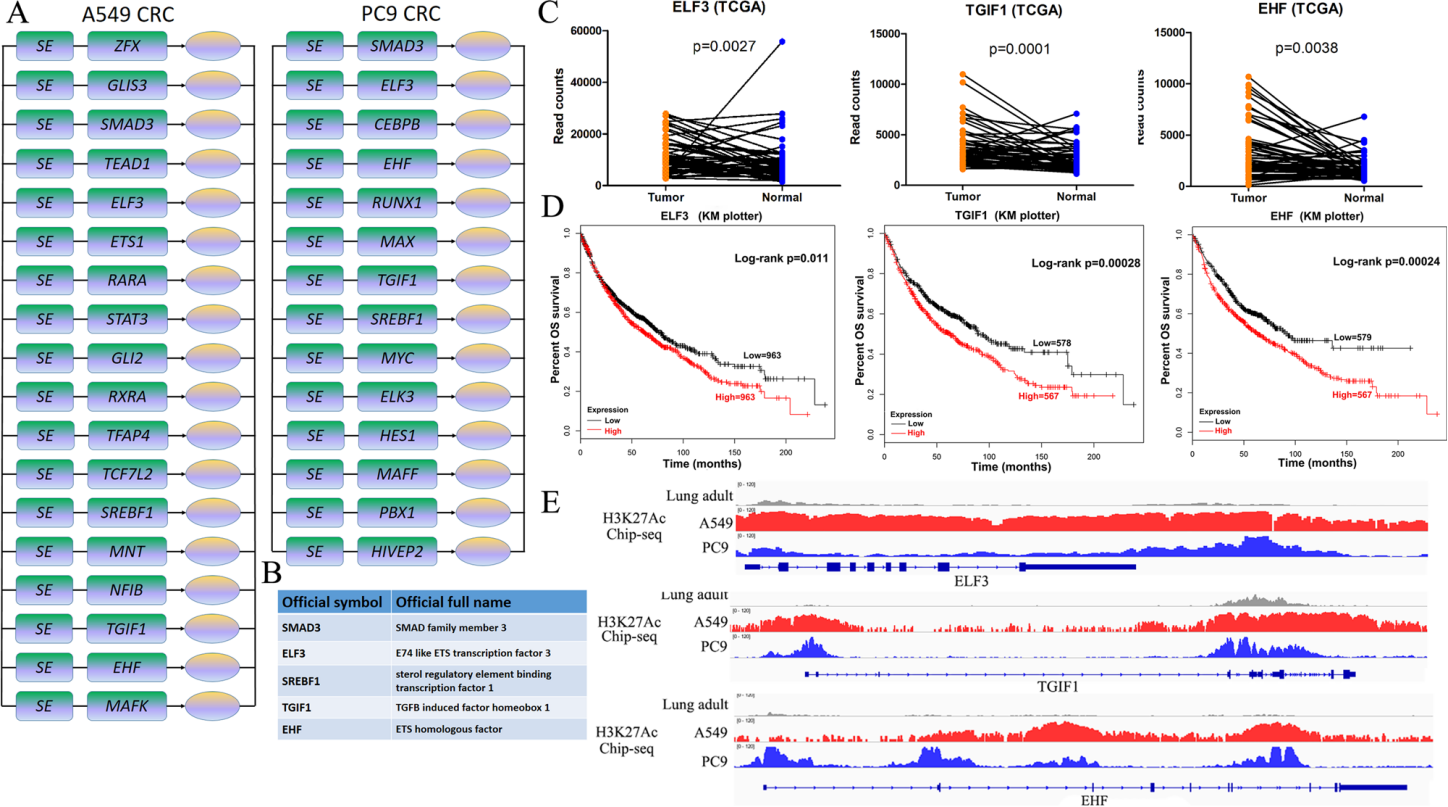
Master TFs in CRC, identified from LUAD-SE genes are frequently overexpressed in human LUAD and are correlated with poor prognosis in LUAD patients. **A** Experimental validation for LUAD cell circuitry. Core regulatory circuit containing ELF3, EHF, and TGIF1 for LUAD cell lines, A549 and PC-9. **B** Intersections of TFs between two individual LUAD cell lines, A549 and PC-9, identified by CRC models. **C** The expression levels of selected TFs in tumor or normal tissues of patients with LUAD based on the TCGA illumina HiSeq RNA-Seq data. The *x*-axis represents the different tissues of LUAD patients and the *y*-axis represents expression read counts of TCGA. **D** Kaplan–Meier survival analysis with TCGA datasets indicates that higher ELF3 (*p* = 0.011), EHF (*p* = 0.00024), and TGIF1 (*p* = 0.00028) expression is associated with a worse overall survival in patients with LUAD. **E** ChIP-seq occupancy profiles of H3K27Ac at the normal adult lung, A549 cell line and PC-9 cell line. H3K27Ac ChIP-seq signal was highly enriched in the ELF3, EHF and TGIF1 genomic loci of A549 and PC-9 cells but not the normal liver. TCGA The Cancer Genome Atlas.

The models of CRC predicted for canonical human LUAD cell lines indicated that the approach described here captures the previously described core TFs and CRC for LUAD and revealed that additional TFs contribute to this core circuitry. We used the A549/PC-9 specific LUAD -SE genes, to reveal the different CRC in different LUAD cell lines. The results showed that 18 TFs contribute to the LUAD CRC in A549 cell line (Fig. 2A, left panel), and 14 TFs in PC-9 cell line (Fig. 2A, right panel). Interestingly, there are five TFs appearing on the intersection between two different LUAD cell lines CRC, including SMAD3, ELF3, SREBF1, TGIF1, and EHF (Fig. 2B). To further determine the roles of these candidate TFs in LUAD, their expression patterns were investigated using data from TCGA. Remarkably, ELF3, EHF, and TGIF1 were highly expressed in LUAD tumor tissue compared with normal lung tissue (Fig. 2C), and the high expression of these TFs were positively correlated with poor prognosis in LUAD patients (Fig. 2D). And these master TFs were confirmed by analysis of H3K27ac ChIP-seq data generated in LUAD cell lines and normal adult lung, showed the occupancy aligned with H3K27ac (Fig. 2E).

These results demonstrate that SE associated master TFs, including ELF3, EHF, and TGIF1, play a prominent role in LUAD progression, through modeling the CRC structure.

### The regulatory interaction network in CRC of LUAD-SE-associated TFs is interdependent

**Fig. 3:**
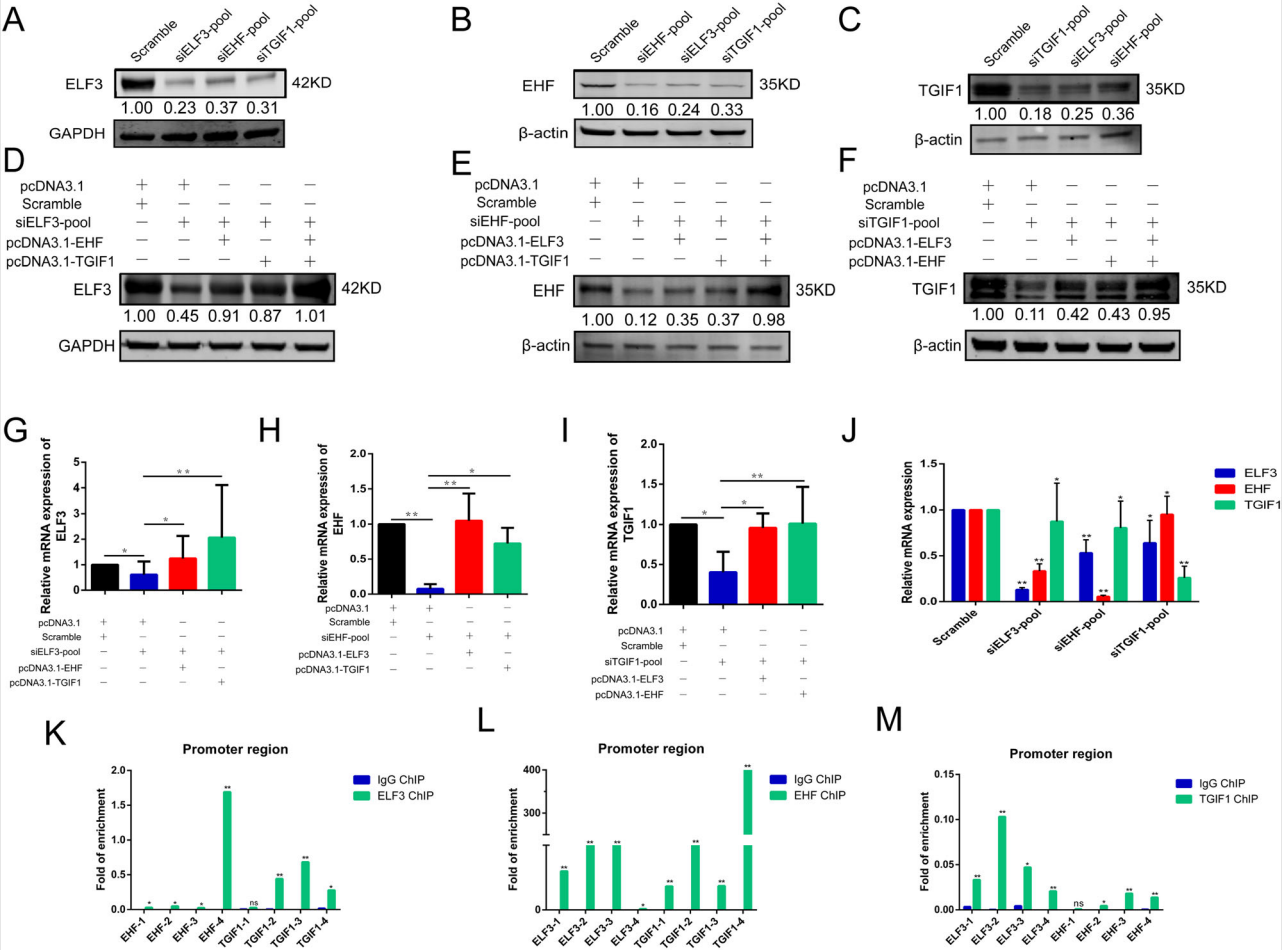
The regulatory interaction network in CRC model of LUAD-SE-associated TFs is interdependent. **A**–**C**, **J** Expressions of all master TFs in the knockdown of any master TF and validating the efficiency of siRNA targeting to master TFs by both real-time PCR and western blot. **D**–**I** Western blot and real-time PCR demonstrating that siRNA co-transfected with pcDNA3.1 could decrease the master TF expression significantly in both protein and mRNA level. This phenomenon could be partly reversed by either of another two master TFs, also could be reversed almost entirely in the combination of another two master TFs. **K**–**M** Data are shown as fold enrichments of master TFs promotor sub-regions in each antibody immunoprecipitate, ELF3 antibody (**K**), EHF antibody (**L**), TGIF1 antibody (**M**), over control IgG immunoprecipitate. GAPDH and β-actin were used as internal control, the lower panels showed the gray scale ratio of protein (ELF3) to GAPDH and protein (EHF and TGIF1) to β-actin. *p < 0.05; **p < 0.01; ***p < 0.001.

We measured the mRNA levels of ELF3, EHF, and TGIF1 in A549 and PC-9 cells by qRT-PCR, and found that these TFs expressed at the average level in PC-9 (Fig. S1a). We designed three individual siRNAs (Table S1) for each master TF, the efficiency of siRNAs and plasmids were analyzed by qRT-PCR and western blot in PC-9 and A549 cells, respectively (Fig. S1B–D). Meanwhile, we constructed overexpression plasmid for each master TF, the efficiency of overexpression plasmids were analyzed by qRT-PCR and western blot in PC-9 cells (Fig. S1E–G). For the best silencing effect, siRNA-pool was transfected in PC-9 and A549 cells. Interestingly, silencing any master TF expression lead to all master TFs expression decreased in protein level (Fig. 3A–C). Similar results were also obtained in A549 cells (Fig. S2A). In PC-9 cells, transfected with any master TFs plasmids result in itself overexpression, and increased expressions of other master TFs at the same time (Fig. S2B–D). Furthermore, we performed rescue experiments in PC-9 with both any master TF silencing and two other master TFs overexpression, resulting in partially, but significantly rescued, by the presence of two other master TFs plasmids separately or together in protein (Fig. 3D–F) and mRNA level (Fig. 3G–J). These results indicated that potential interconnections may be involved in CRC constructed by ELF3, EHF, and TGIF1 in LUAD.

To validate direct transcriptional regulation among these TFs, we investigated the binding of each master TF to the other two master TFs promotor region by ChIP-qPCR assay. The promotor region of each master TF was divided into four subregions (-1, -2, -3, and -4) and we analyzed the binding of TFs to promotor sub-region by qPCR (Fig. 3K–M). Strikingly, each master TFs could bind promotor regions other than itself, implying an interconnected circuitry may be formed, as we had predicted and observed.

Collectively, our data confirmed the existence of CRC constructed by ELF3, EHF, and TGIF1 in LUAD. Each master TF has dual functions, regulating itself transcription and as a component of core master TFs to maintain interior equilibrium via CRC.

### ELF3, EHF, and TGIF1 enhances the malignant phenotypes of LUAD cells

**Fig. 4:**
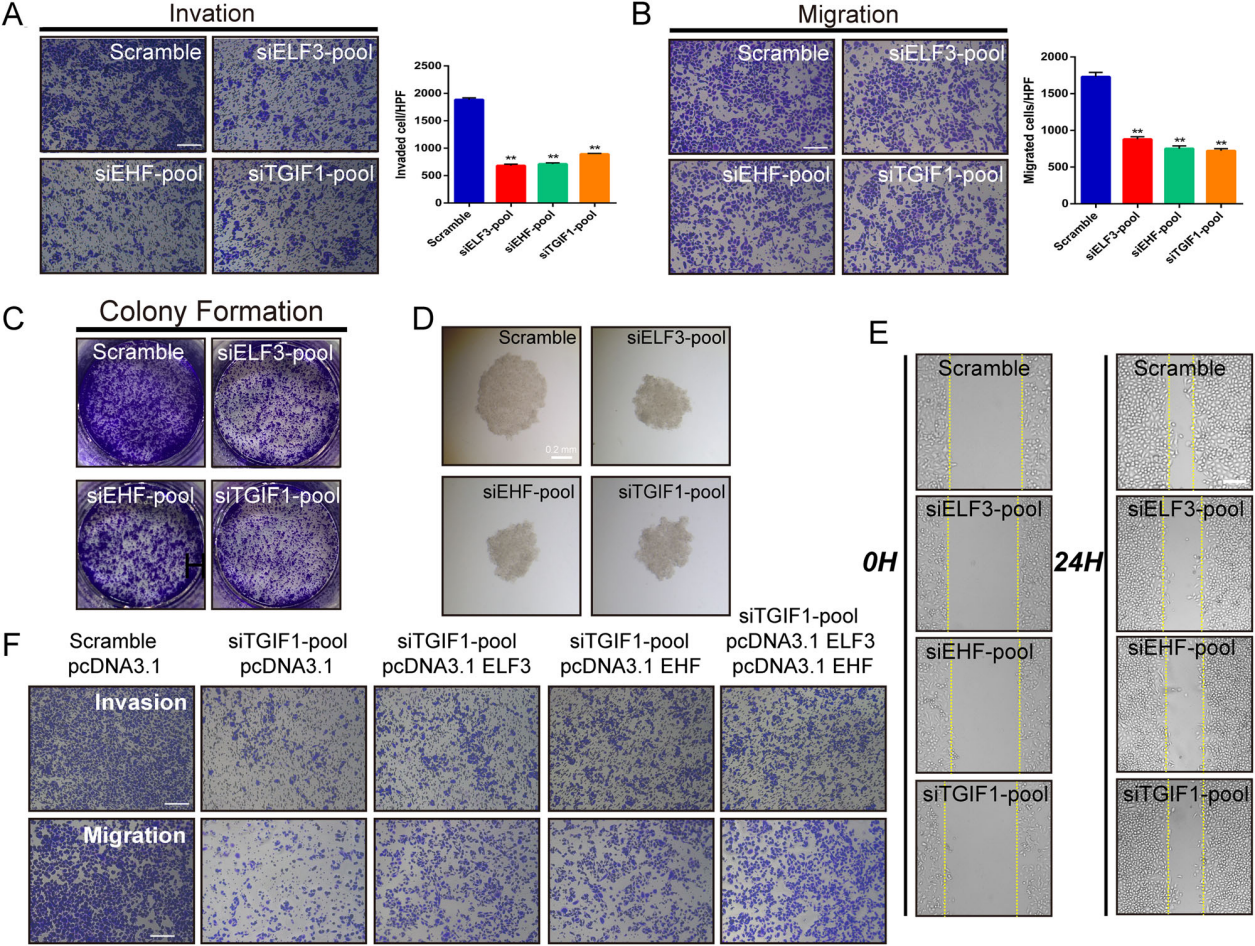
ELF3, EHF, and TGIF1 enhances the malignant phenotypes of LUAD cells. **A**, **B** Inhibition of invasive and migrate activity by knockdown master TFs, ELF3, EHF, and TGIF1. PC-9 cells were transfected with indicated siRNA-pool and subjected to invasion and migration assays (see “Materials and methods”). Invaded and migrated cells were stained with crystal violet and counted (see Fig. S3). Histograms represent the number of invasion or migration. Representative photographs were shown. **C** Colony assay performed on PC-9 cells transfected with either scramble or siRNA-pool targeting ELF3, EHF, and TGIF1. **D** Silencing of ELF3, EHF, and TGIF1 increased clonal cell growth in a 3D spheroid culture system. PC-9 cells transfected with scramble or siRNA-pool were cultured as spheroid in DMEM medium and the spheroids was observed after 48 h. **E** Inhibition of cell migration by knockdown master TFs, ELF3, EHF, and TGIF1. PC-9 cells were transfected with indicated siRNA-pool and subjected to scratch assay (see “Materials and methods”). Representative photographs were taken and migration areas were measured. Statistical analysis was performed by using *t* tests; data are shown as bar graphs of the mean ± SD. of three independent experiments. Scale bars (white) indicate 250 μm. **F** The effects of pcDNA3.1-ELF3 and/or pcDNA3.1-EHF co-transfected with TGIF1 siRNA on cell migration and invasiveness in LUAD cells were examined by Transwell assays using a Boyden chamber in the presence or absence of Matrigel, respectively. Histograms represent the number of invasion or migration. Data were representative of two to three separate experiments performed in triplicate. *p < 0.05; **p < 0.01; ***p < 0.001.

We set out to elucidate the function of ELF3, EHF, and TGIF1 in LUAD. The effect of master TFs on LUAD cell was determined next. In both Transwell assay and Matrigel invasion assay, cell migratory and invasive capabilities were enhanced by forced expression, and were significantly decreased by silencing of master TFs. (Fig. 4a, b). Similar results were found in the plate clone-forming assay, in which the colony number of LUAD cells was decreased with master TFs silencing (Fig. 4C; Fig. S3A). In A549 cells, capability of cell invasion and migration were also decreased significantly when silencing ELF3, EHF, and TGIF1 (Fig. S3B). Spheroid assays were performed using hanging-droplet plates and non-adherent U-bottom 96-well plates. We grew spheroids of PC-9 cells transfected with scramble or siRNA-pool. Notably, we consistently observed that knockdown of ELF3, EHF, and TGIF1 hardly formed large well-formed spheroids in each well (Fig. 4D). Results showed that treatment with siRNA had significant effect on the LUAD spheroids, and it significantly reduced the volumes of LUAD spheroids (Fig. S3C). In addition, cell migration of PC-9 cells in the absence of ELF3, EHF, and TGIF1 were significantly inhibited (Fig. 4E; Fig. S3D). For concreteness, we take the TGIF1 as an example to investigate if mutual regulations among master TFs in CRC affect LUAD cells malignant phenotype. Consistent with our previous observations, TGIF1 silencing resulted in LUAD cells malignant phenotype attenuation which could be partially and significantly rescued by overexpression of ELF3 and/or EHF (Fig. 4F; Fig. S3E, F).

These data together suggest that SE associated master TFs, ELF3, EHF, and TGIF1, plays a prominent role in promoting tumor cells malignant progression, through core regulatory network by constructing CRC in LUAD.

### Inhibition of the SE-associated key targets attenuated LUAD malignant progression via the suppression of master TFs in CRC

**Fig. 5:**
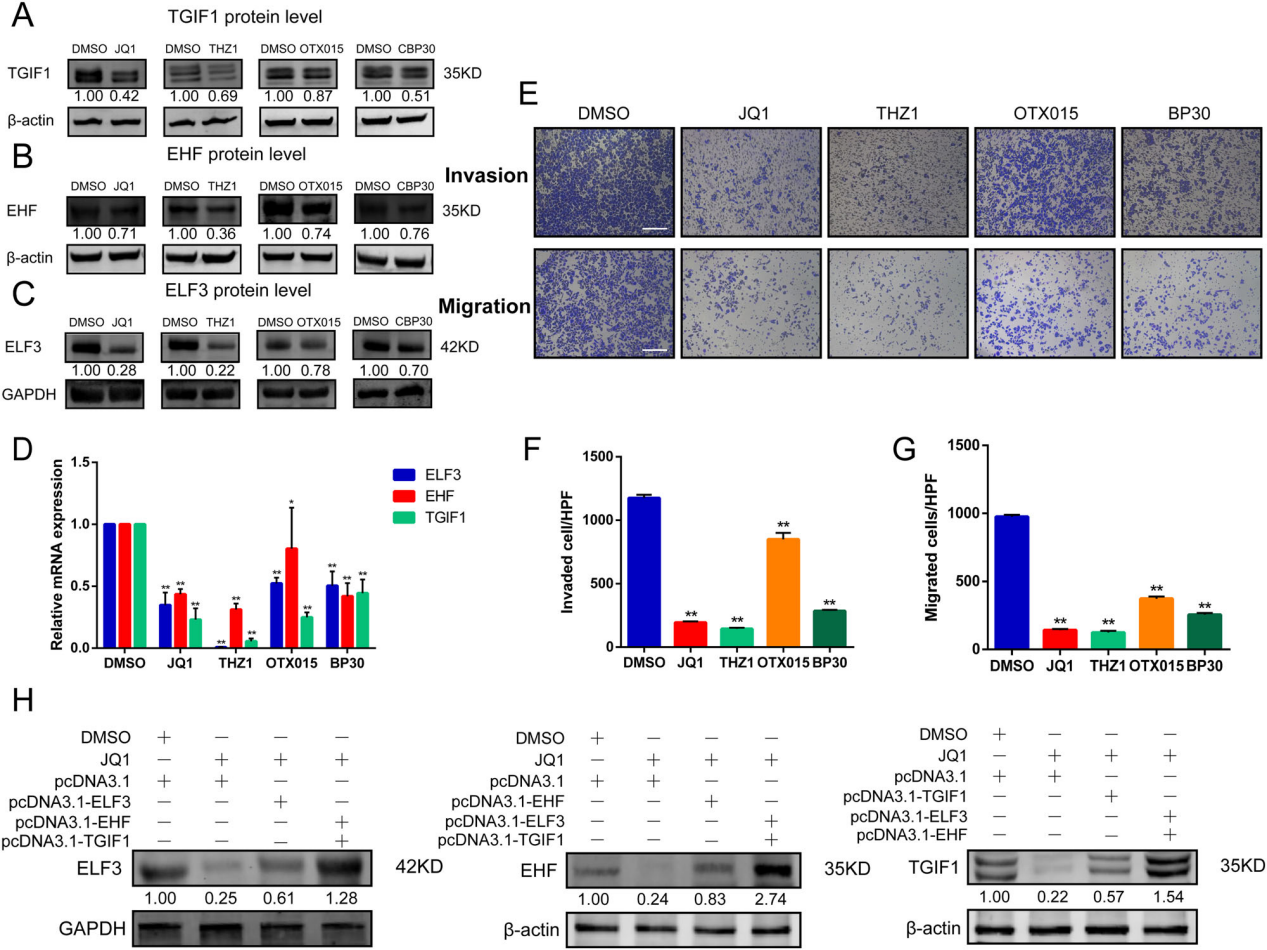
Inhibition of the SE-associated key targets attenuated LUAD malignant progression via the suppression of master TFs in CRC model. **A**–**C** Perturbation of SE associated key targets, including BRD4, EP300, and CDK7, by small-molecule inhibitors reduced the expression of master TFs significantly at protein levels. **D** Expressions of master TFs in LUAD cell line PC-9 treated with SE associated targets inhibitors in mRNA level were analyzed by real-time PCR. **E** Inhibition of invasive and migrate activity by perturbation of SE associated key targets via small molecular inhibitions. PC-9 cells were cultured with indicated small molecular inhibitions and subjected to invasion and migration assays (see “Materials and methods”). Invaded and migrated cells were stained with crystal violet and counted. Representative photographs were shown. **F**, **G** Histograms represent the number of invasion or migration cells. **H** In PC-9 cell lines, alterations of ELF3, EHF, and TGIF1 at protein levels while JQ1 treated for 100 nM 48 h together with or without transfected with overexpression plasmids. GAPDH and β-actin were used as internal control, the lower panels showed the gray scale ratio of protein (ELF3) to GAPDH and protein (EHF and TGIF1) to β-actin. *p < 0.05; **p < 0.01; ***p < 0.001.

CDK7, BRD4, and EP300, key components of SE complex, are highly enriched in SEs, where they synergistically activate the SE-associated genes including master TFs in CRC. Therefore, we hypothesized inhibition of SE complex components could affect master TFs resulting in attenuating LUAD malignant progression. According to the characteristics of these components, we employed small molecular inhibitors in currently tested in clinical trials, such as JQ1 (BRD4 inhibitor), OTX015 (BRD inhibitor), CBP30 (EP300 inhibitor), and THZ1 (CDK7 inhibitor). Upon addition of these inhibitors to LUAD cells to repress all master TFs expression in both protein (Fig. 5A–C) and mRNA level (Fig. 5D). In A549 cells, expression of ELF3, EHF, and TGIF1 also decreased when small molecular inhibitors addition compared to DMSO addition (Fig. S3F). In both Transwell assay and Matrigel invasion assay, cell migratory, and invasive capabilities were decreased by inhibition of these key SE complex components (Fig. 5E), Histogram representing the number of invasive or immigrate cells per 200× field (Fig. 5F, G). These results revealed that targeted small molecular inhibitors could suppress LUAD cells malignant progression via perturbation of SE complex key components resulting in master TFs decreased and CRC structure destroyed. Furthermore, we performed rescue experiments in PC-9 cells in the presence of JQ1, transfected with or without any master TF overexpression plasmid. Results showed that expression of ELF3, EHF, and TGIF1 in the presence of JQ1 were significantly increased by others overexpression. These suggested that ELF3, EHF, and TGIF1 could be promoted by each other and protected each other from disturbance of SE (Fig. 5H).

### High expression of CRC (ELF3/EHF/TGIF1-High) were associated with poor prognosis on patients with LUAD

**Fig. 6:**
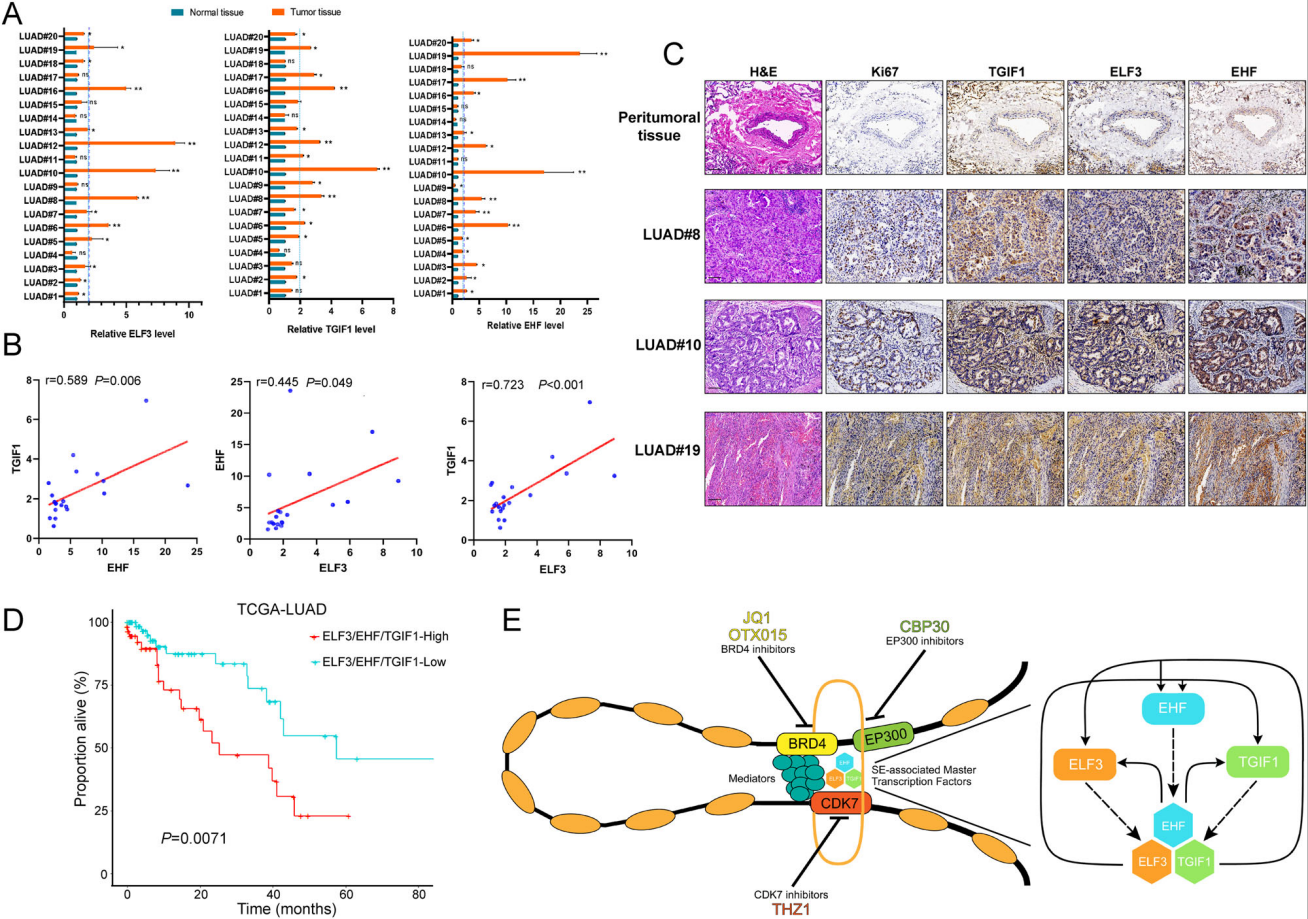
High expression of CRC (ELF3/EHF/TGIF1-High) were associated with poor prognosis on patients with LUAD. **A** qRT-PCR analysis of ELF3, EHF, and TGIF1 among 20 pairs of matched lung adenocarcinoma and adjacent normal tissue. **B** Correlation analyses conducted between the mRNA expression of ELF3, EHF, and TGIF1 from (**A**). **C** ELF3, EHF, and TGIF1 were detected in peritumoral tissue and representative LUAD samples by immunohistochemistry staining. Scale bar, 100 μm. **D** Kaplan–Meier analysis of overall survival of LUAD patients (TCGA-LUAD) with low (low expression of ELF3, EHF, and TGIF1, *n* = 78) and high (high expression of ELF3, EHF, and TGIF1, *n* = 52) ELF3/EHF/TGIF1 expression (log-rank test, two-sided). **E** Left panel: a schematic diagram showing the key targets and small-molecule inhibitors of SE. JQ1 and OTX015 targeting BRD4, THZ1 targeting CDK7; CBPC30 targeting EP300. Right panel: CRCs are assembled as fully interconnected loops of auto-regulated TFs. Schematic graph of the model of interconnected circuitry, with hexagons representing enhancer elements and proteins, respectively. Data in (**A**) were mean ± SD; *n* = 3 independent experiments, two-tailed paired Student’s *t* test, ns not significant, *p < 0.05; **p < 0.01; ***p < 0.001 (**B**) R, Pearson correlation coefficients (*r*) and *p* values.

Following the identification and characterization of the core transcriptional circuitry composed by ELF3, EHF, and TGIF1, we next focused on investigating the biological implications of CRC on patients with LUAD. To examine if ELF3, EHF and TGIF1 levels are important in human cancers, we measured mRNA expression of these in LUAD tissues along with their paired adjacent peritumoral tissues. There was differential expression between peritumoral and cancerous tissues in the majority of cases, with half tumors expressing at least twofold higher levels of ELF3, EHF, and TGIF1 (Fig. 6A). Correlation analyses also revealed a positive relationship between ELF3, EHF, and TGIF1 (Fig. 6B). Furthermore, we performed serial sections on the peritumoral tissue and LUAD tissue, which attempted to confirm if high expression of CRC was associated with tumor progression. Ki-67 staining was performed to visualize proliferating cells, indicating high proliferative activity of the tumor. IHC revealed that expressions of ELF3, EHF and TGIF1 were higher in LUAD tumor tissues compared with peritumoral tissues (Fig. 6C). And this gene expression signature may be reflective of high “proliferative” activity. To confirm association between master TFs expression and patient survival, we categorized gene expression as low or high in comparison with the median value: if the expression level was higher than the median, it was classified as high, whereas if it was lower than the median. Interestingly, high CRC levels, simultaneous high expression of ELF3, EHF, and TGIF1 (ELF3/EHF/TGIF1-High, *n* = 52), were also predictive of poor overall survival compared to low expression of CRC (ELF3/EHF/TGIF1-Low, *n* = 78) in TCGA-LUAD dataset (Fig. 6D). These results corroborated biological implications of CRC composed by ELF3, EHF, and TGIF1 in promoting tumor progression, which would be extremely clinically valuable.

These data suggest a model (Fig. 6E, left panel) where the majority of LUAD SEs are associated with master TFs CRC (Fig. 6E, right panel) involving ELF3, EHF, and TGIF1, which with mediators provide a strong transcriptional complex for downstream oncogenes to accelerating aggravation of LUAD progression. However, in the presence of targeted small molecular inhibitors, SE complex become vulnerable, which is followed by master TFs decreased and CRC structure destroyed, eventually leading to attenuate LUAD progression.

## Discussion

Despite numerous new insights gained from genomic analyses of patients with LUAD, preventive, or therapeutic strategies have not substantially improved outcomes. LUAD exhibits high intertumour and intratumour genomic heterogeneity, increasing the barriers to exploiting targetable genomic lesions. Alternative molecular approaches, especially transcriptional regulatory mechanisms, in addition to genomic profiling are essential to decipher LUAD malignant tumor progression for the development of more innovative and effective regimens. The gene expression program is vigorously rewired during the multistep malignant transformation of LUAD. Accurate interactions at genomic regulatory elements, such as the SEs, between TFs, co-factors and chromatin regulators have been shown to effect the transcriptional progression.

To this end, we established and functionally validated an interconnected transcriptional circuitry formed by SE associated master TFs (ELF3, EHF, and TGIF1), which orchestrates the dysregulation of LUAD transcriptome in a cooperative, synergistic, and complementary manner. By interacting with their SEs, these master TFs promote the expression of each other. Indeed, both their protein and mRNA levels correlate with each other significantly and are generally high in LUAD tumors compared with normal adult lung tissues. Because of this pivotal role of master TFs in the LUAD transcriptional regulation (especially via controlling SEs), all of these are required for the survival and malignant progression of LUAD cells.

ELF3, a documented tumor suppressor in many epithelial tumors, displays strong prognostic value in LUAD and has been proved to be an oncogene and putative therapeutic target in LUAD^27^. In NSCLC, ELF3 has been identified to be a potential oncogenic TF by genome-wide identification^28^. MIR-320a-3p/ELF3 axis could regulate cell metastasis and invasion via PI3K/Akt pathway, and overexpression of ELF3 facilitates cell growth and metastasis also through PI3K/Akt and ERK signaling pathways^29,30^. In hepatocellular carcinoma, ELF3 promotes epithelial-mesenchymal transition by protecting ZEB1 from miR-141-3p-mediated silencing^31^. Mutation alteration of ELF3 is associated with a characteristic elevation in the expression of immune checkpoint molecules in biliary tract cancer^32^, and has been proved to be the promising candidate for targeted in distal cholangiocarcinoma, ampullary carcinoma^33^.

EHF is a member of the ETS TF family that has been implicated as a tumor suppressor for prostate cancer and a regulator of inflammation in airway epithelium^34^. As target gene of BRG1, EHF has been shown to contribute to differentiation and proliferation of epithelial cells, as well as EZH2 expression, and apoptotic signaling regulation in prostate cancer stem cells^35^. We find that upregulating of EHF could promote LUAD cell migration and invasion, while downregulation suppressed LUAD cell migratory and invasive ability. These results suggest that EHF has a cancer-promoting effect in LUAD and is a promising novel target for gene therapy.

TGIF1 belongs to the superfamily of TALE homeodomain proteins, which control an array of important cellular processes, such as proliferation, differentiation, and apoptosis. Although TGIF1 has been shown as tumor suppressor in pancreatic ductal adenocarcinoma (PDAC)^36,37^, our finding demonstrated that TGIF1 plays a role as tumor enhancer in LUAD cells^38^. In breast cancer, TGIF1 also behave like oncogene promoting migration, invasion and metastasis in MDA-MB-231 human breast cancer cells^39^. The function of TGIF1 in LUAD was strikingly different from PDAC. We can only speculate about the reasons for this discrepancy. These discrepancies are mainly due to tumor heterogeneity and different tumor types. As such, our identification of TGIF1 as a potential oncogene in LUAD that interacts with ELF3 and EHF provides an unprecedented SE-associated CRC model for future identification and investigation of these potential targets amenable to therapeutic intervention in LUAD.

Transcriptional dependency represents the Achilles heel of cancer cells. The last key step to build CRC is that CRCs are assembled as fully interconnected loops of autoregulated TFs (Fig. 6E). In the present study, we demonstrated that LUAD cells are vulnerable exceptionally to perturbation of SE-associated key targets. Such an “Achilles heel” of pathogenesis could be exploited to develop new treatment therapies. Theoretically, SE-associated key target inhibition would be more effective in patients who overexpress components of the SE-associated CRC model. There is a compelling evidence that the ability of LUAD PC-9 cells to invade and migrate induced by interconnected transcriptional circuitry (ELF3, EHF, and TGIF1) is decreased upon treatment with SE-associated targets, inhibitors such as JQ1, THZ1, OTX015, and CBP30. Thus, these observations suggest that these small-molecule inhibitions could function as tumor suppressor indirectly dependent on SE-associated master TFs CRC in LUAD.

Our pan-cancer analyses from TCGA revealed that aberrant expressions of ELF3, EHF, and TGIF1 are prevalent in a wide spectrum of cancers (Fig. S4A–C) and high levels of these master TFs are associated with poor patient outcomes (Fig. 2D). Meanwhile, pan-cancer analysis data showed the same trend as gene expression profile across all tumor samples and paired normal tissues from GEPIA (Fig. S4A–C). According to the gene expression profile from TCGA, we could obtain reliable correlation between the three master TFs. There was a positive correlation between ELF3 with EHF and TGIF1, but EHF was negatively correlated with TGIF1 (Fig. S4D). After bioinformatics analysis of gene expression profiles, the expression of master TFs were further identified via the qRT-PCR method. The results suggested that after transfected with siRNA individually in PC-9 cell line, these master TFs showed different degrees of decrease in mRNA level (Fig. S4E). We can only speculate as to why our results diverge from prior findings. We ascribe such inconsistency to two main factors. The first one is that protein expression level in pan-cancer from TCGA do not represent the true situation of that in LUAD. The second one is that the different correlation between EHF and TGIF1 in mRNA expression level and pan-cancer analysis maybe caused by post-transcriptional modification. These may hint at ELF3 playing a pivotal role in the CRC model function. The present study also evaluated the relationship between different expression levels and the prognosis of patient with LUAD, respectively. Compared with ELF3 and EHF, the high expression of TGIF1 could be considered as an warning sign of patient poor prognosis (Fig. S5). However, the TGIF1, even the CRC model, in clinical practice they are of little value until now their utility could be clearly demonstrated and validated in our study.

In summary, this is the first comprehensive study on the SE-associated master TFs CRC transcriptional control in LUAD. We demonstrated a novel transcriptional regulation model in LUAD, which is SE associated master TFs CRC including ELF3, EHF, and TGIF1. All of the components of master TFs CRC could promote tumor progression in LUAD. Interaction of the CRC model is cooperative, synergistic, and complementary. The represent study indicated that, although each of master TFs we identified has a same role in promoting LUAD progression, yet their emphasis differs. ELF3, as a bridge linking EHF and TGIF1, is of vital importance in CRC interaction. TGIF1, may be a better prognostic biomarker for patient with LUAD. Although the CRC model playing a powerful role in LUAD tumor progression, focusing on the origin of CRC model, SE-associated master TFs, provide us new therapeutic targets for LUAD treatment.

## Supplementary information

Supplementary data

Supplementary Figure S1

Supplementary Figure S2

Supplementary Figure S3

Supplementary Figure S4

Supplementary Figure S5

Supplementary Table S1

Supplementary Table S2

Supplementary Table S3

Supplementary Table S4
