# Supplementary material for: Aberrant super-enhancer landscape reveals core transcriptional regulatory circuitry in lung adenocarcinoma
Source: Oncogenesis. 2020 Oct 17;9(10):92. doi: 10.1038/s41389-020-00277-9 (PMC7568720; doi:10.1038/s41389-020-00277-9)
Supplement: Supplementary file 1 — Supplementary data [file 41389_2020_277_MOESM1_ESM.pdf]

Aberrant super-enhancer landscape reveals core transcriptional regulatory circuitry in lung adenocarcinoma

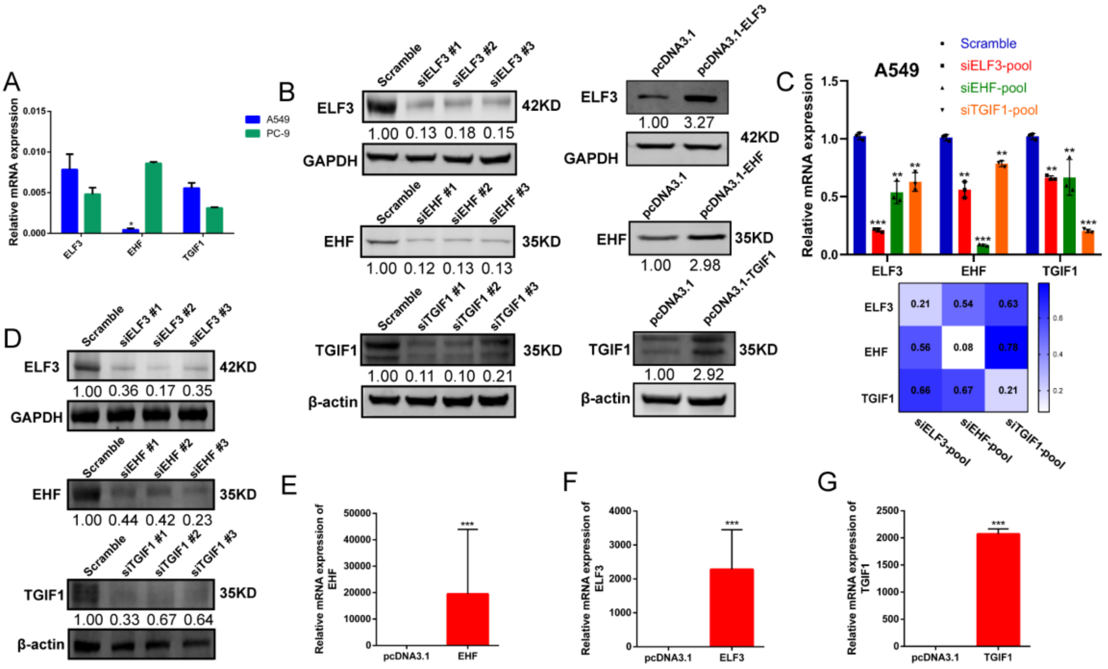

Figure S1. (A) Expressions of ELF3, EHF and TGIF1 in A549 and PC-9 cell lines. (B) Overexpress and knockdown efficiency were assessed by western blot. GAPDH and β-actin were used as internal control, the lower panels showed the gray scale ratio of protein (ELF3) to GAPDH and protein (EHF and TGIF1) to β-actin. (C) Up panel: In A549 cells, expression of all master TFs in the knockdown of any master TF and validating the efficiency of siRNA targeting to master TFs by real-time PCR. Down panel: The correlation of RNA expression between EHF, ELF3 and TGIF1. (D) In A549 cells, knockdown efficiency of siRNAs were assessed by western blot. (E-G) Overexpress efficiency were assessed by qRT-PCR. GAPDH and β-actin were used as internal control, the lower panels showed the gray scale ratio of protein (ELF3) to GAPDH and protein (EHF and TGIF1) to β-actin. *, $p<0.05$; **, $p<0.01$; ***, $p<0.001$

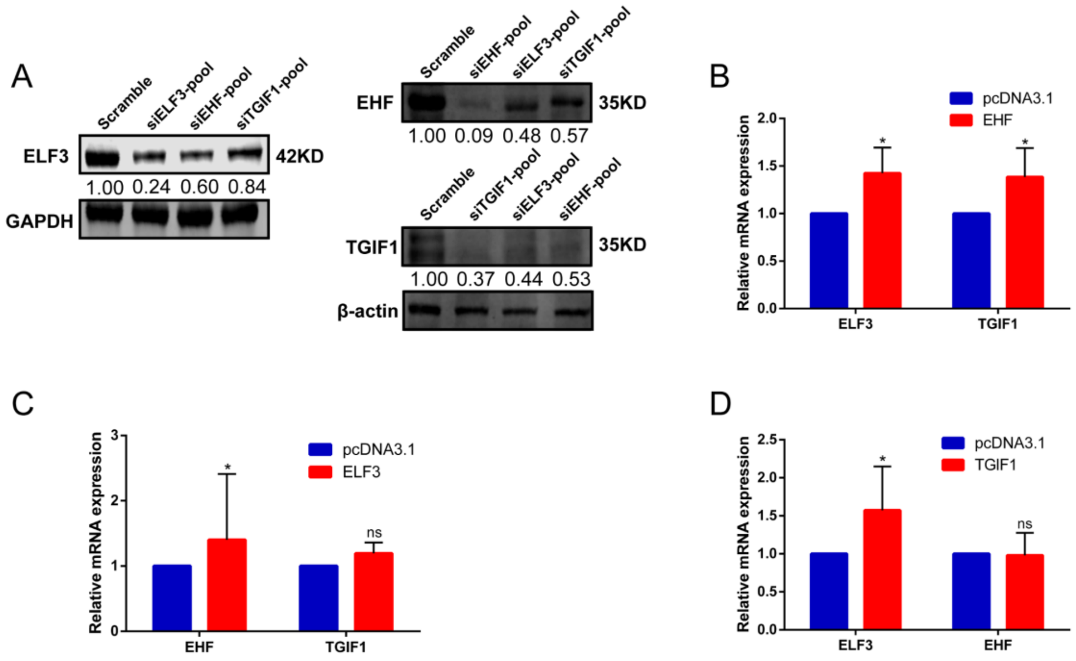

Figure S2. (A) Expression of all master TFs in the knockdown of any master TF and validating the efficiency of siRNA targeting to master TFs by western blot in A549 cells. (B-D) Each of the master TFs overexpression increases the mRNA levels of the other two master TFs to certain extent. *, $p<0.05$; **, $p<0.01$; ***, $p<0.001$.

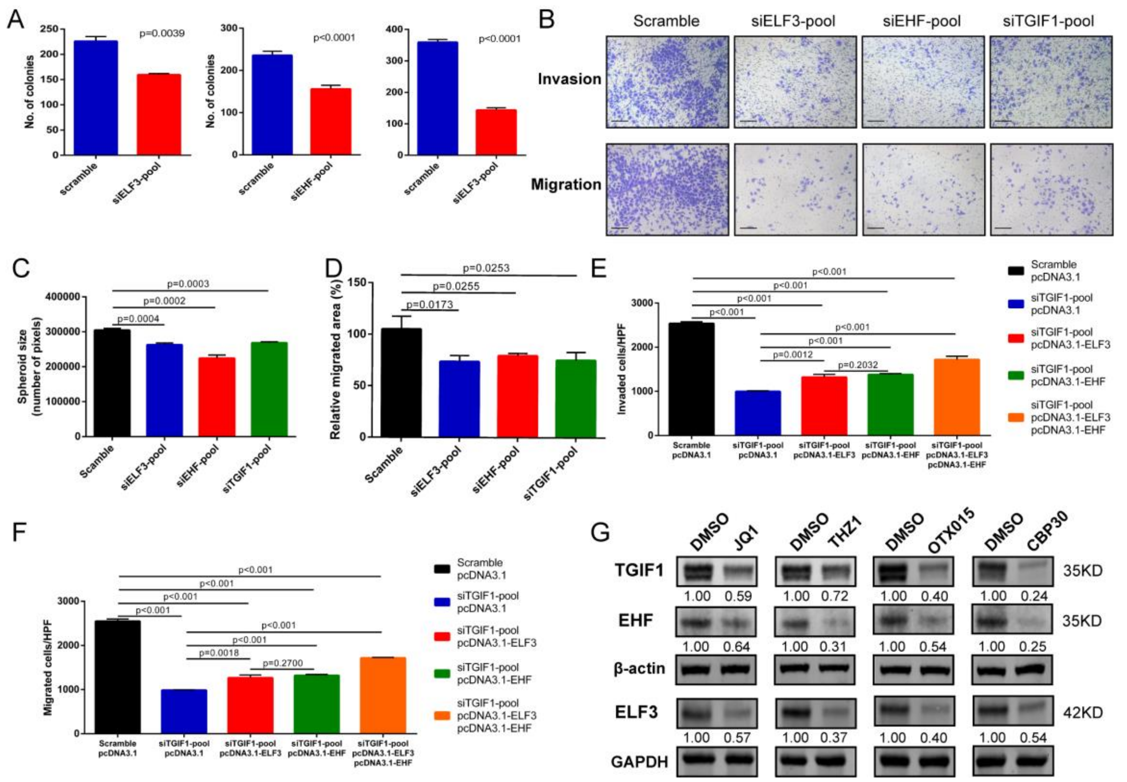

Figure S3. (A) As a supplement of Figure 4C, histograms represent the number of colonies. (B) Inhibition of invasive and migrate activity by knockdown master TFs, ELF3, EHF and TGIF1. A549 cells were transfected with indicated siRNA-pool and subjected to invasion and migration assays (see Materials and Methods). Invaded and migrated cells were stained with crystal violet. Representative photographs were shown. (C) As a supplement of Figure 4D, histograms represent the spheroid size (number of pixels). (D) As a supplement of Figure 4E, histograms represent the percentage of migrated area after scratch wound. (E, F) As a supplement of Figure 4F, histograms represent the number of invaded or migrated cells/HPF. (G) In A549 cells, perturbation of SE associated key targets, including BRD4, EP300 and CDK7, by small-molecule inhibitors reduced the expression of master TFs significantly at protein levels. GAPDH and β-actin were used as internal control, the lower panels showed the gray scale ratio of protein (ELF3) to GAPDH and protein (EHF and TGIF1) to β-actin. *, $p<0.05$; **, $p<0.01$; ***, $p<0.001$

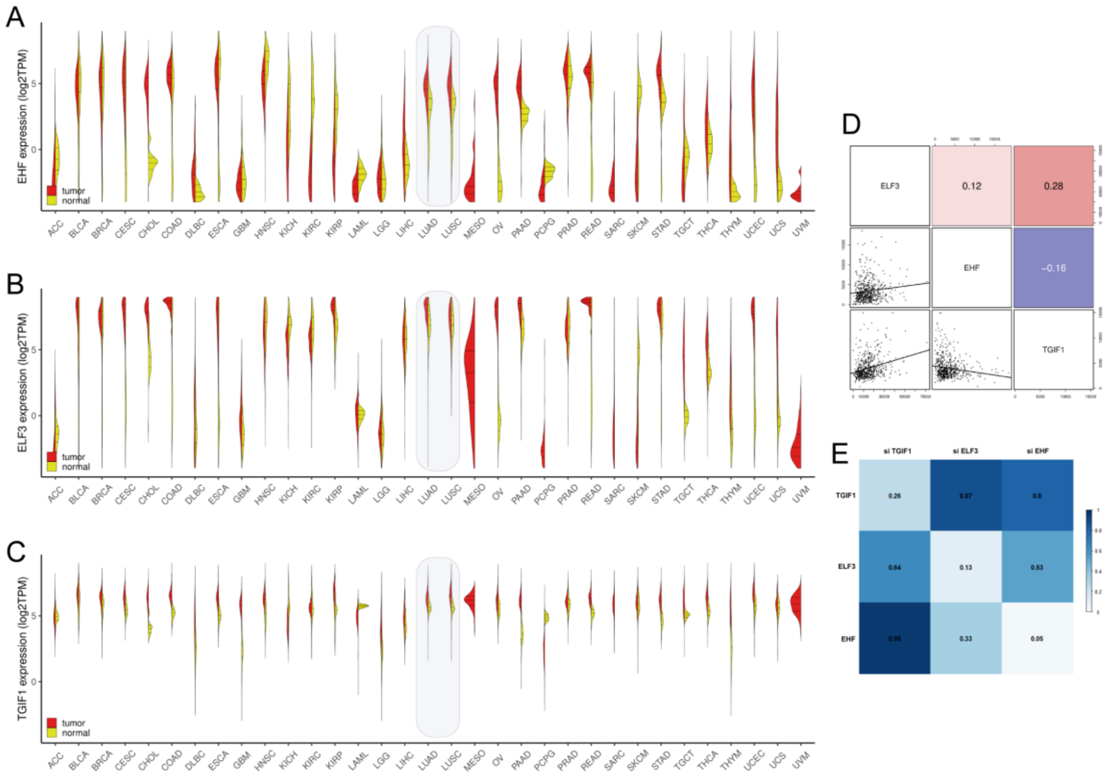

Figure S4. Pan-cancer analysis of ELF3, EHF and TGIF1. (A-C) Pan-cancer analysis of EHF, ELF3 and TGIF1 expressions across cancers from TCGA. (D) The correlation of protein expression profiles from TCGA between EHF, ELF3 and TGIF1. The protein expressions of EHF and TGIF1 were positive correlated with ELF3. (E) The correlation of RNA expression between EHF, ELF3 and TGIF1, data of (A-D) were from (Gene Expression Profiling Interactive Analysis, GEPIA) GEPIA 2 (http://gepia2.cancer-pku.cn/)

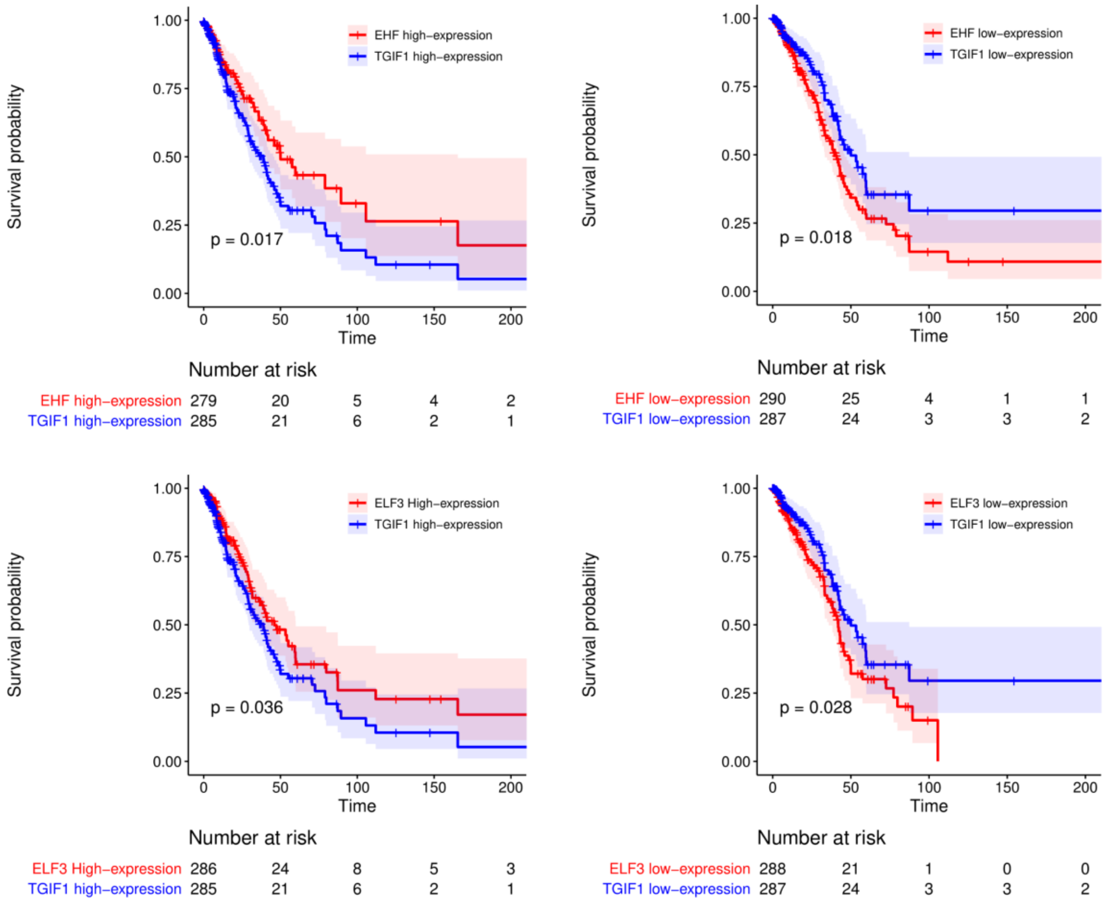

Figure S5. Survival curves were generated by Kaplan-Meier survival analysis. Kaplan-Meier curves indicating the overall survival of well-defined LUAD cases derived from Kaplan-Meier Plotter (http://kmplot.com/analysis/) with high or low expression of ELF3, EHF and TGIF1.
