# Supplementary figures and images for: Aberrant super-enhancer landscape reveals core transcriptional regulatory circuitry in lung adenocarcinoma
Source: Oncogenesis. 2020 Oct 17;9(10):92. doi: 10.1038/s41389-020-00277-9 (PMC7568720; doi:10.1038/s41389-020-00277-9)

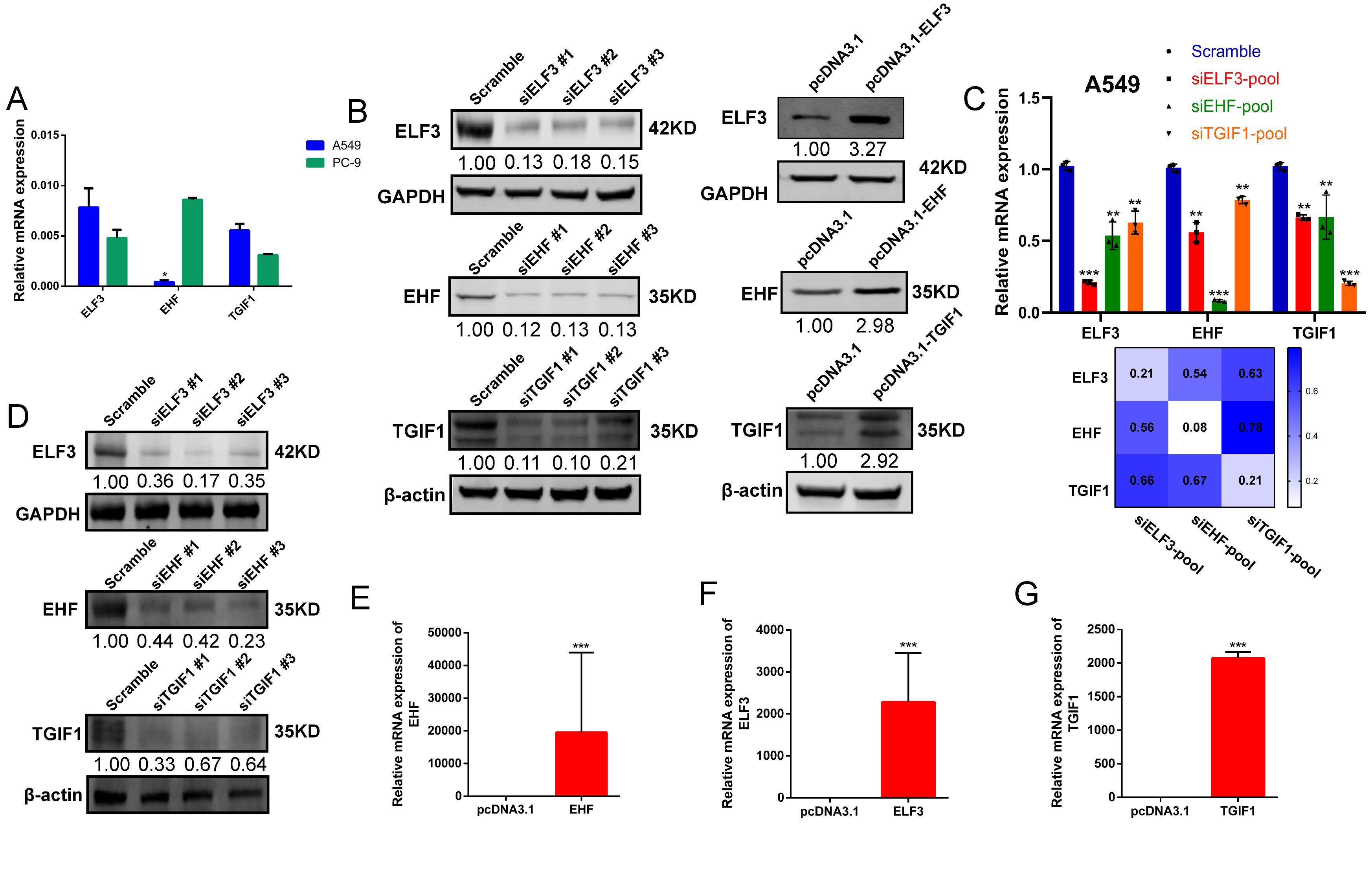

Supplement: Supplementary file 2 — Supplementary Figure S1 [file 41389_2020_277_MOESM2_ESM.tif]

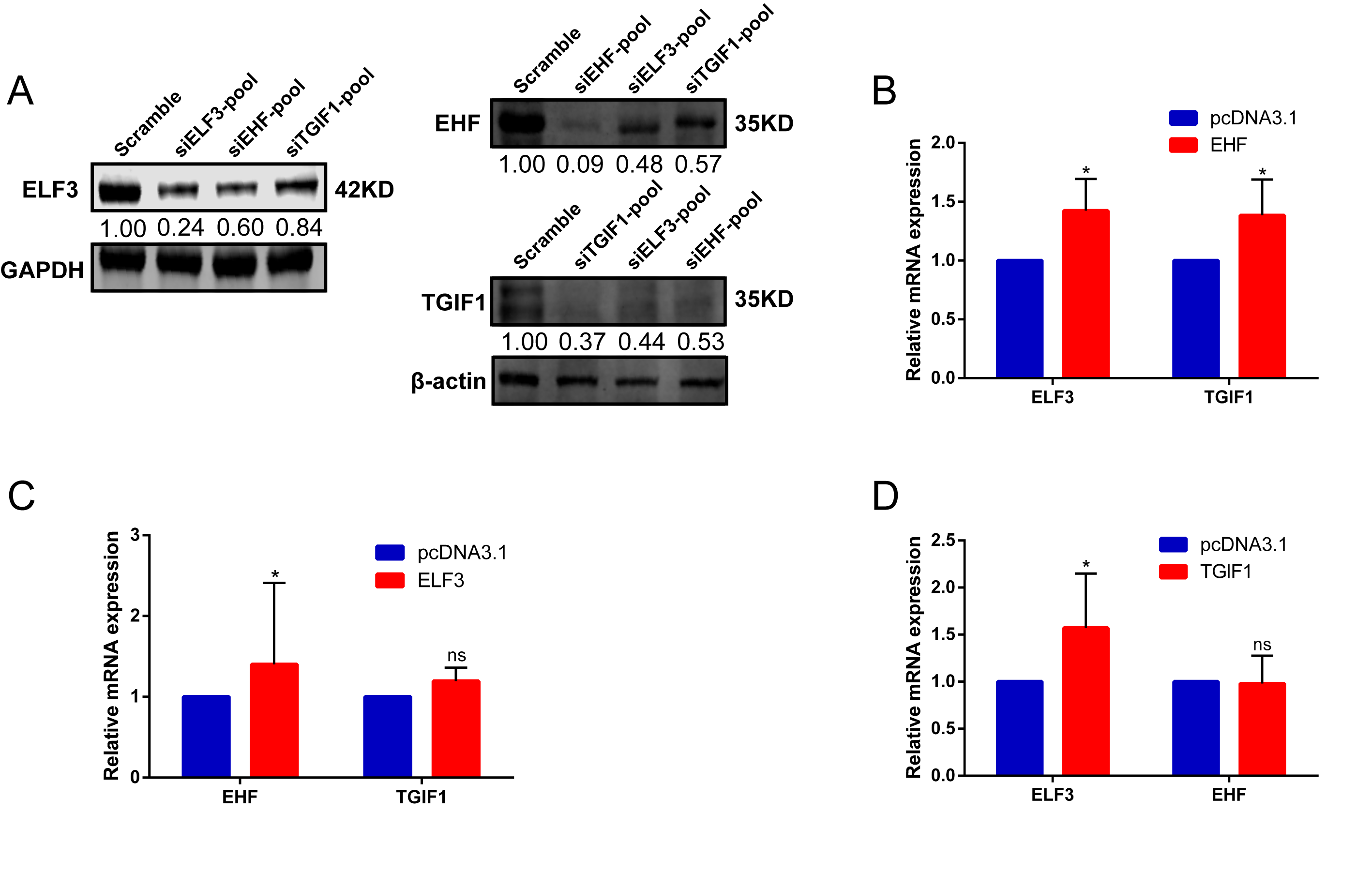

Supplement: Supplementary file 3 — Supplementary Figure S2 [file 41389_2020_277_MOESM3_ESM.tif]

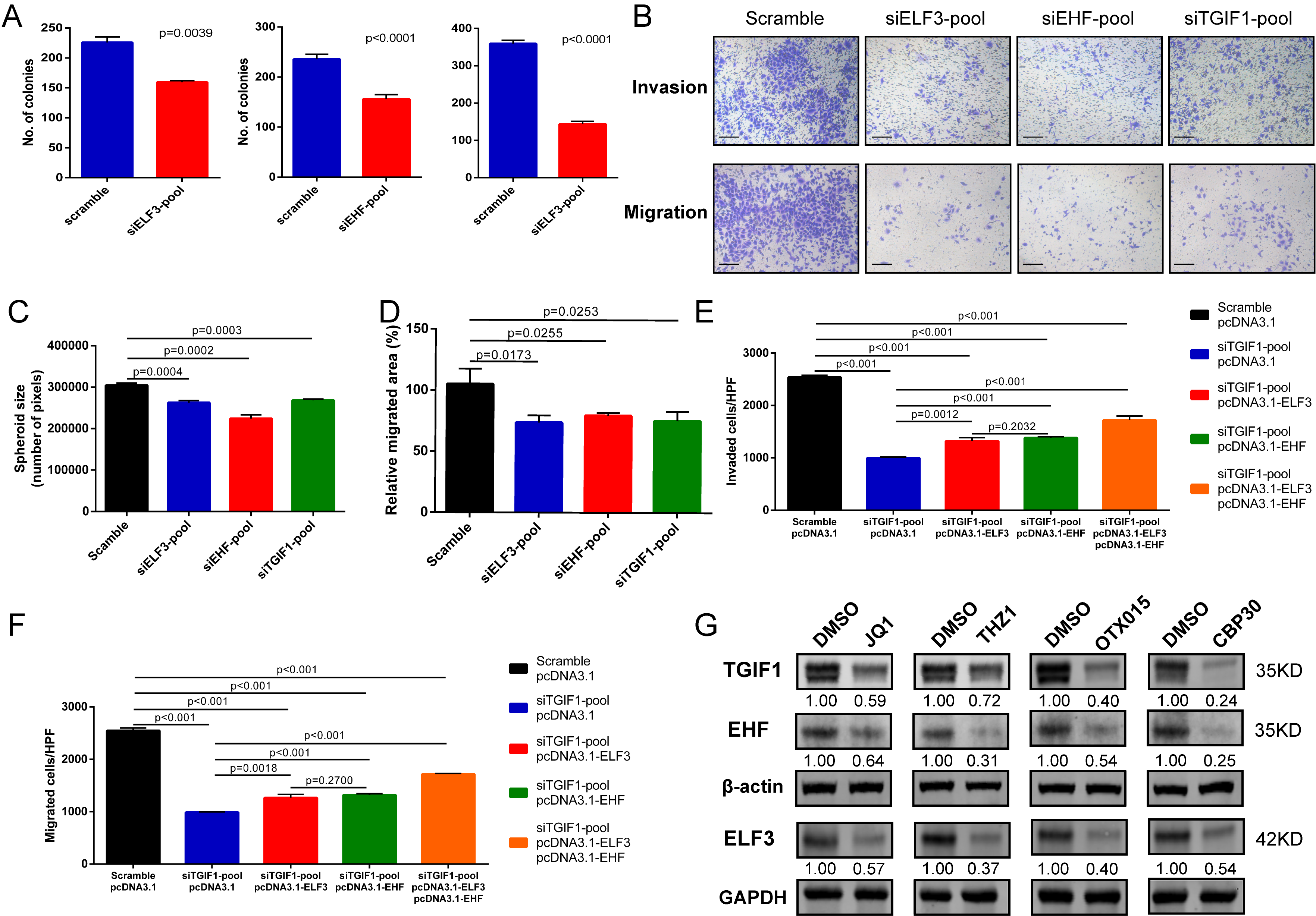

Supplement: Supplementary file 4 — Supplementary Figure S3 [file 41389_2020_277_MOESM4_ESM.tif]

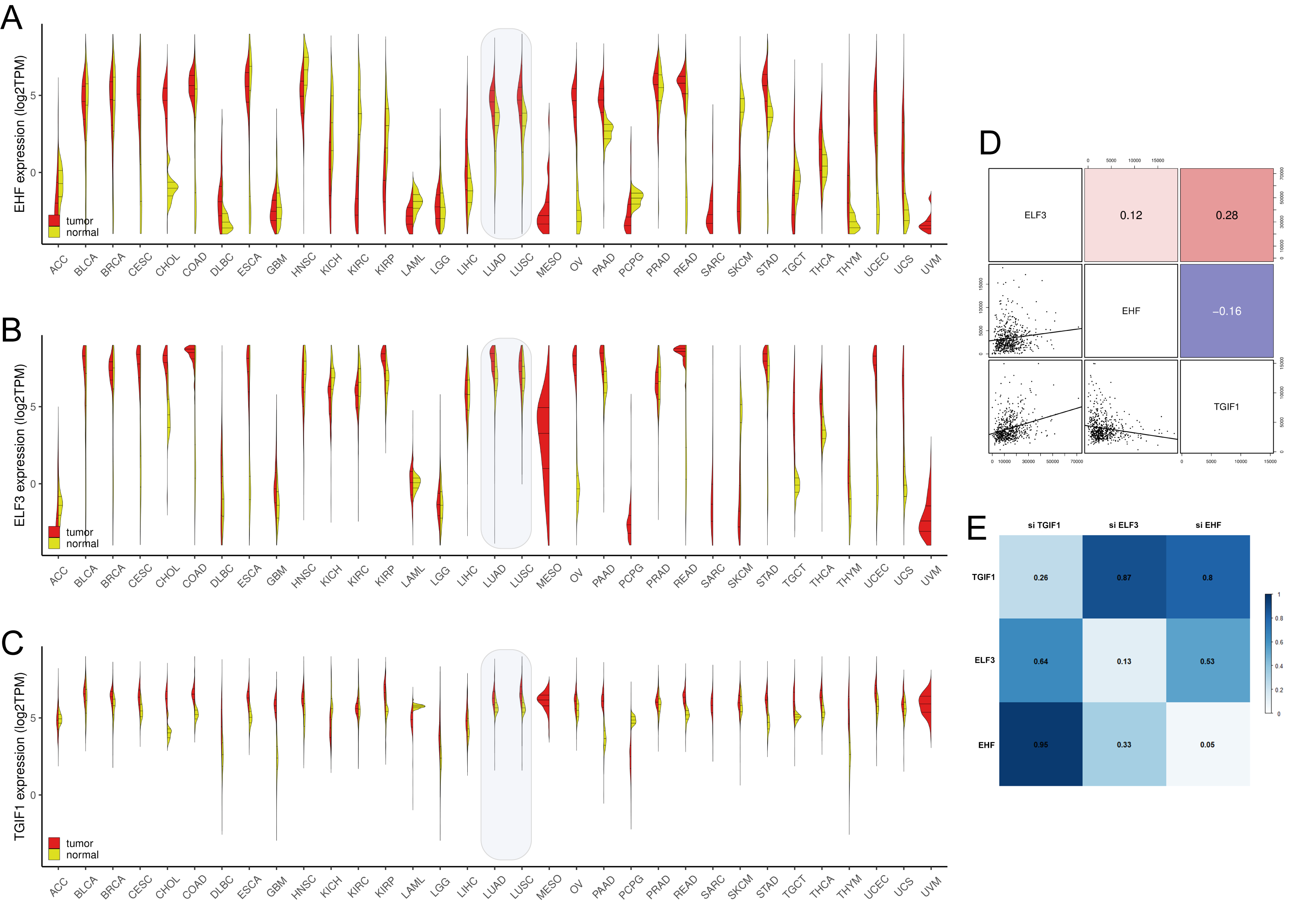

Supplement: Supplementary file 5 — Supplementary Figure S4 [file 41389_2020_277_MOESM5_ESM.tif]

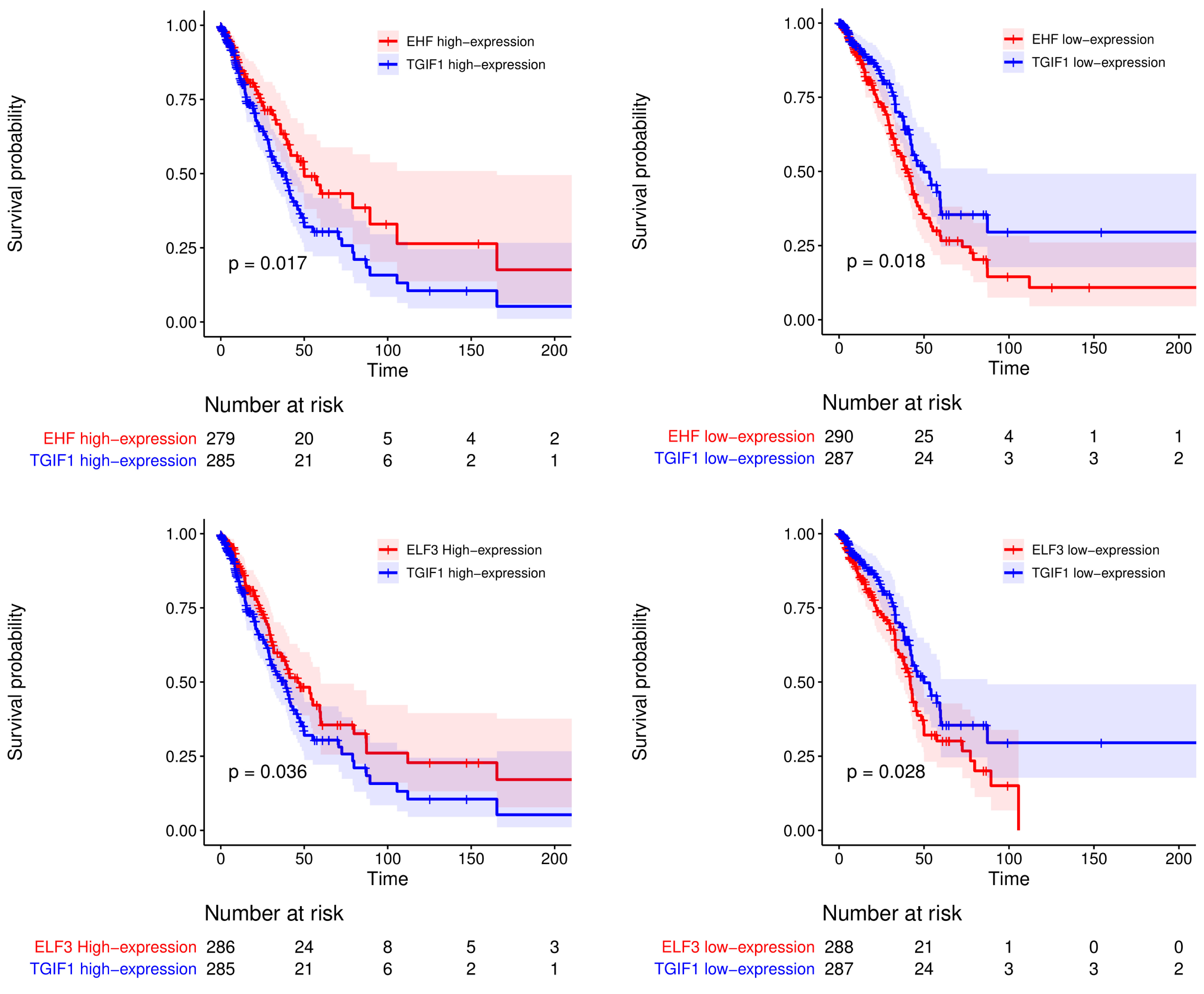

Supplement: Supplementary file 6 — Supplementary Figure S5 [file 41389_2020_277_MOESM6_ESM.tif]
