# Supplementary material for: Aberrant super-enhancer landscape reveals core transcriptional regulatory circuitry in lung adenocarcinoma
Source: Oncogenesis. 2020 Oct 17;9(10):92. doi: 10.1038/s41389-020-00277-9 (PMC7568720; doi:10.1038/s41389-020-00277-9)
Supplement: Supplementary file 7 — Supplementary Table S1 [file 41389_2020_277_MOESM7_ESM.pdf]

Supplementary Table S1 Sequences of siRNA oligonucleotides

Oligo set	Sequence (sense)
si-ELF3#1	5'-GCUGCAACCUGUGAGAUUA-3'
si-ELF3#2	5'-CC-UCUGCAAUUGUGCCCUU-3'
si-ELF3#3	5'-CCAUGAGGUACUACUACAA-3'
si-EHF#1	5'-GCCAGUGGCAUGAAAUUCATT-3'
si-EHF#2	5'-CCACACACAAUGUCAUUGUTT-3'
si-EHF#3	5'-CAGCCGAGCUAUGAGAUAUTT-3'
si-TGIF1#1	5'-GCAUUGAAAGAUGUCCCUUTT-3'
si-TGIF1#2	5'-GGACAUUCCCUUGGACCUUTT-3'
si-TGIF1#3	5'-CCAGAGGACACUUGUAAAUTT-3'

ELF3, E47 like epitelial-sepecific transcription factor 3; EHF, epithelial-sepecific homologous factor; TGIF1, transforming growth factor beta induced factor homeobox 1
