# Supplementary material for: Aberrant super-enhancer landscape reveals core transcriptional regulatory circuitry in lung adenocarcinoma
Source: Oncogenesis. 2020 Oct 17;9(10):92. doi: 10.1038/s41389-020-00277-9 (PMC7568720; doi:10.1038/s41389-020-00277-9)
Supplement: Supplementary file 8 — Supplementary Table S2 [file 41389_2020_277_MOESM8_ESM.pdf]

Supplementary Table S2 Oligonucleotide sets used for construct

Oligo set	Primers	Sequence
pcDNA3.1-ELF3	Forward	5'-CTAGCGTTTAAACTTAAGCTTGCCACCatggctgcaacctgtgagattagc-3'
	Reverse	5'-GCCCTCTAGACTCGAGCGGCCGCtcagttccgactctggagaacctc-3'
pcDNA3.1-EHF	Forward	5'-CTAGCGTTTAAACTTAAGCTTGCCACCatgattctggaaggaggtggtgta-3'
	Reverse	5'-GCCCTCTAGACTCGAGCGGCCGCtcagttttcattttctctccatcc-3'
pcDNA3.1-TGIF1	Forward	5'-CTAGCGTTTAAACTTAAGCTTGCCACCatggacattcccttggacctttct-3'
	Reverse	5'-GCCCTCTAGACTCGAGCGGCCGCttaagctgtaagttttgcctgaag -3'

ELF3, E47 like epitelial-sepecific transcription factor 3; EHF, epithelial-sepecific homologous factor; TGIF1, transforming growth factor beta induced factor homeobox 1
