# Supplementary material for: Aberrant super-enhancer landscape reveals core transcriptional regulatory circuitry in lung adenocarcinoma
Source: Oncogenesis. 2020 Oct 17;9(10):92. doi: 10.1038/s41389-020-00277-9 (PMC7568720; doi:10.1038/s41389-020-00277-9)
Supplement: Supplementary file 9 — Supplementary Table S3 [file 41389_2020_277_MOESM9_ESM.pdf]

Supplementary Table S3 Primer sets used for RT-PCR and ChIP-PCR

Primer set	Primers	Sequence	Application
ELF3	Forward	5'-CACTGATGGCAAGCTCTTC-3'	RT-PCR
	Reverse	5'-GGAGCG-CAGGAACTTGAAG-3'	
EHF	Forward	5'-TGCAGCATCTGAAGTGGAAC-3'	RT-PCR
	Reverse	5'-AGGAAGGTGACTGGTGGTTG-3'	
TGIF1	Forward	5'-GGATTGGCTGTATGAGCACCGT-3'	RT-PCR
	Reverse	5'-GCCATCCTTTCTCAGCATGTCAG-3'	
ELF3 Promotor sub-region 1 (ELF3-1)	Forward	5'-TAGAGTTAAAGAAAGAAGTATCTGTAGAAGGTGGAGT-3'	ChIP-PCR
	Reverse	5'-AGTCTCGCTCTGTCGCCCA-3'	
ELF3 Promotor sub-region 2 (ELF3-2)	Forward	5'-CCACCTCAAAACAAAATAAACAAAATACTAGATCT-3'	ChIP-PCR
	Reverse	5'-TGGTTATTCCCATTTTATAGACATGAAAGTTGC -3'	
ELF3 Promotor sub-region 3 (ELF3-3)	Forward	5'-TGCATTCCTTACAGGACTTTTTTGGT-3'	ChIP-PCR
	Reverse	5'-CAGGGGCTGGAAGGTGCTC-3'	
ELF3 Promotor sub-region 4 (ELF3-4)	Forward	5'-GCCAGAGTCCTGGGTGTTCT-3'	ChIP-PCR
	Reverse	5'-GGAGCTCCTCCCTGAGCTCA-3'	
EHF Promotor sub-region 1 (EHF-1)	Forward	5'-ATTAAAGGTGATTGGGACTGGGTCA-3'	ChIP-PCR
	Reverse	5'-TTCCCACCCTTCCCAACTGT-3'	
EHF Promotor sub-region 2 (EHF-2)	Forward	5'-TGTCTGGGGCAGGTTAGGGA-3'	ChIP-PCR
	Reverse	5'-TGCTCCTAGCAAAGAAAAATACCATCC-3'	
EHF Promotor sub-region 3 (EHF-3)	Forward	5'-GTCATTCACCCGTATGGGAGAAGGT-3'	ChIP-PCR
	Reverse	5'-AAGGGGCTCCCAGCAAGTTC -3'	
EHF Promotor sub-region 4 (EHF-4)	Forward	5'-CAGAGCCTGGTACTTATAGAGCT-3'	ChIP-PCR
	Reverse	5'-GAAGATAAATGCATATAACAATAATTTTAAATCATTTTGT-3'	
TGIF1 Promotor sub-region 1 (TGIF1-1)	Forward	5'-CCAGAATGTTTGCAGAGCCTCC-3'	ChIP-PCR
	Reverse	5'-GACATCAGCTTTTGGGTACAGAGT-3'	
TGIF1 Promotor sub-region 2 (TGIF1-2)	Forward	5'-AGACTCTCTGCTTTCAACACCAAGTA-3'	ChIP-PCR
	Reverse	5'-TCACACCTGTAATCCCAGCAATTTG-3'	
TGIF1 Promotor sub-region 3 (TGIF1-3)	Forward	5'-GCCTCCGTGCCTGGCCA-3'	ChIP-PCR
	Reverse	5'-ATGGGGCATTGCCTCTGAGAC-3'	
TGIF1 Promotor sub-region 4 (TGIF1-4)	Forward	5'-GCCTGCCTGATTACTGTTTGCT-3'	ChIP-PCR
	Reverse	5'-ACTCCCGGAAGGAGGAGCG-3'	
β-actin	Forward	5'-TGCCCATCTACGAGGGGTATG-3'	ChIP-PCR
	Reverse	5'-TCTCCTTAATGTCACGCACGATTT-3'	RT-PCR
GAPDH	Forward	5'-CCATGGGGAAGGTGAAGGTC-3'	ChIP-PCR
	Reverse	5'-GAAGGGGTCATTGATGGCAAC-3'	RT-PCR

ELF3, E47 like epitelial-sepecific transcription factor 3; EHF, epithelial-sepecific homologous factor; TGIF1, transforming growth factor beta induced factor homeobox 1; GAPDH, glyceraldehyde 3-phosphate dehydrogenase
