# Supplementary material for: Aberrant super-enhancer landscape reveals core transcriptional regulatory circuitry in lung adenocarcinoma
Source: Oncogenesis. 2020 Oct 17;9(10):92. doi: 10.1038/s41389-020-00277-9 (PMC7568720; doi:10.1038/s41389-020-00277-9)
Supplement: Supplementary file 10 — Supplementary Table S4 [file 41389_2020_277_MOESM10_ESM.pdf]

Supplementary Table S4 ELF3, EHF and TGIF1 expression in human lung adenocarcinoma tissues

Clinicopathologic Factor	Total	ELF3 expression		EHF expression		TGIF1 expression	
	n	n	*P*-value	n	*P*-value	n	*P*-value
**Age (years)**							
≤60	10	4	0.178	5	0.256	4	0.371
>60	10	7		7		6	
**Gender**							
Male	9	5	0.964	6	0.343	7	0.142
Female	11	6		5		5	
**Tumor size (diameter)**							
≤3 cm	10	3	0.178	6	0.640	5	0.653
>3 cm	10	6		7		6	
**Lung tumor location**							
Central	7	3	0.887	5	0.658	3	0.639
Peripheral	13	6		8		7	
**Distant metastasis**							
Negative	15	3	0.015	2	0.005	3	0.002
Positive	5	4		4		5	
**TNM stage**							
I/II	12	3	0.028	4	0.018	2	0.009
III/IV	8	6		7		6	

TNM, tumor-node-metastasis
